# Supplementary figures and images for: Aneuploidy influences the gene expression profiles in Saccharomyces pastorianus group I and II strains during fermentation
Source: PLoS Genet. 2022 Apr 7;18(4):e1010149. doi: 10.1371/journal.pgen.1010149 (PMC9032419; doi:10.1371/journal.pgen.1010149)

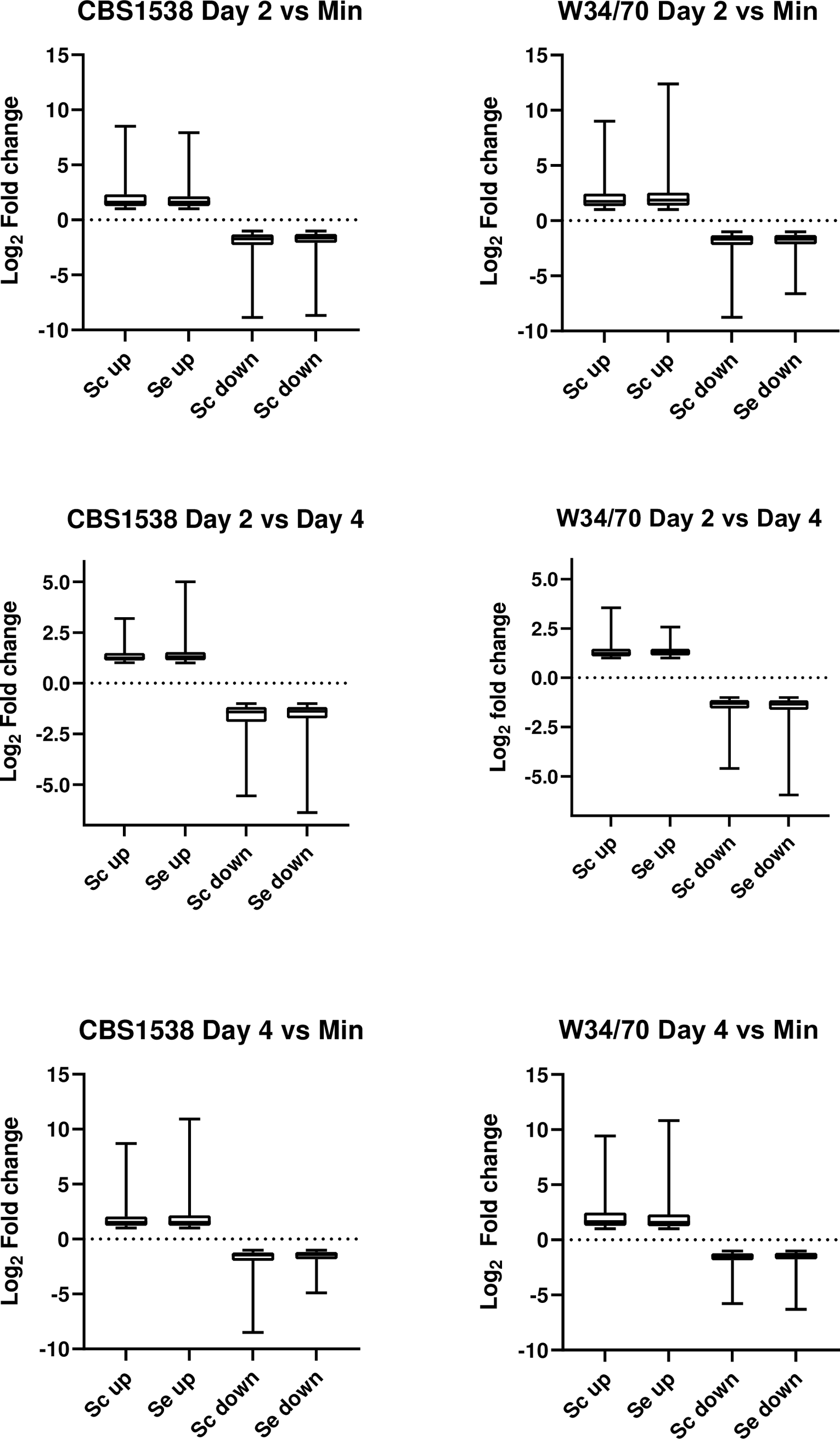

Supplement: S1 Fig — The box extends from the 25th to 75th percentiles, the mean is shown by a bar within the box. The whiskers show the minimum and maximum values in the data set. (TIF) [file pgen.1010149.s001.tif]

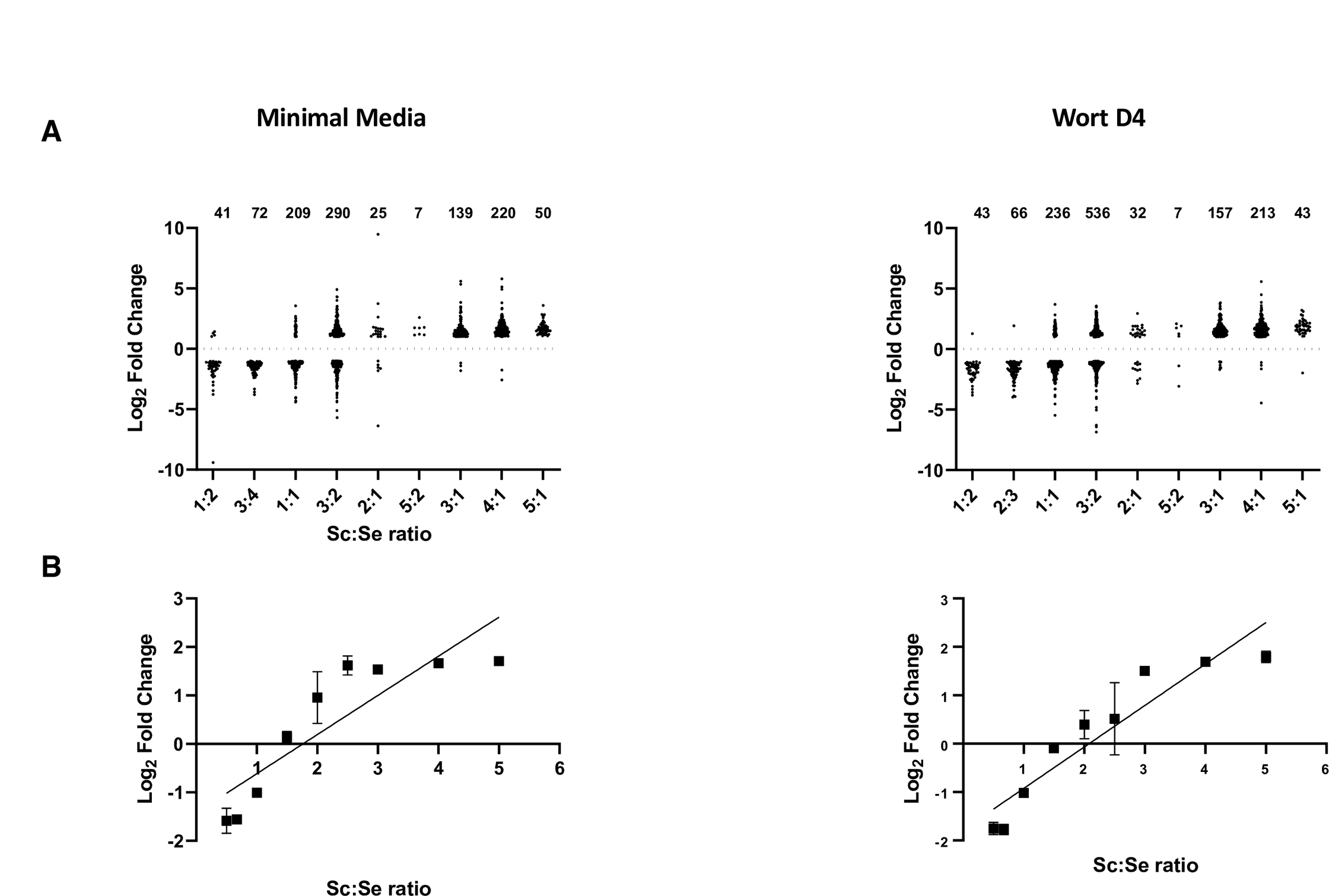

Supplement: S2 Fig — The DEG of Sc to Se orthologues were grouped according to the Sc to Se ratio of gene copy number in W34/70 (A) and CBS1538 (D). Data for minimal medium and for Day 4 (D4) are shown. The number of paired orthologues at each copy number ratio is shown above the graphs. B. Correlation of differential expression of Sc:Se gene orthologues in W34/70 with gene orthologue copy number ratio. The mean of log2 fold changes in expression between Sc and Se orthologues is plotted against Sc:Se gene orthologue copy number ratio. Error bars represent the standard error of the mean. C. Log2-fold gene expression difference between Sc and Se orthologues on chromosome X in W34/70. The dotted line marks the recombination site (THD2) on the hybrid chromosome. Sc:Se ratio to left of vertical dotted line is 3:1 and after 1:1. (TIF) [file pgen.1010149.s002.tif]

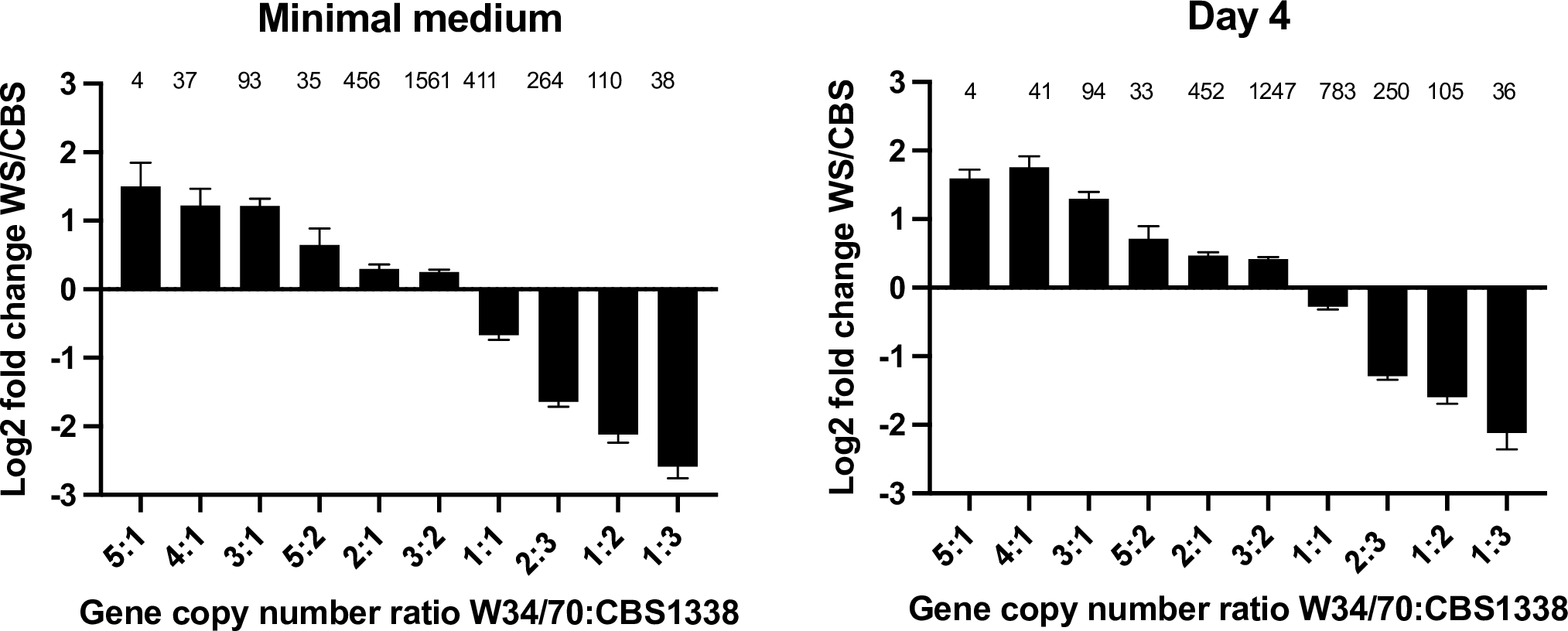

Supplement: S3 Fig — The ratio of the gene copy number for the sum of all alleles (Sc+Se) was compared to Log2-fold differences in gene expression in W34/70 and CBS1538. The number of genes in each ratio category is shown above the bars. Data for Day 4 in wort and for growth in minimal medium is shown. The error bars represent the standard error of the mean Log2-fold change in gene expression between WS34/70 and CBS1538 strains. (TIF) [file pgen.1010149.s003.tif]
